# Thousands of Novel Endolysins Discovered in Uncultured Phage Genomes

**DOI:** 10.3389/fmicb.2018.01033

**Published:** 2018-05-18

**Authors:** Iris Fernández-Ruiz, Felipe H. Coutinho, Francisco Rodriguez-Valera

**Affiliations:** Evolutionary Genomics Group, Departamento de Producción Vegetal y Microbiología, Universidad Miguel Hernández de Elche, San Juan de Alicante, Spain

**Keywords:** bacteriophage, endolysins, metagenomics, biotechnology, protein discovery

## Abstract

Bacteriophages express endolysins toward the end of their replication cycle to degrade the microbial cell wall from within, allowing viral progeny to be released. Endolysins can also degrade the prokaryotic cell wall from the outside, thus have potential to be used for biotechnological and medical purposes. Multiple endolysins have been identified within the genomes of isolated phages, but their diversity in uncultured phages has been overlooked. We used a bioinformatics pipeline to identify novel endolysins from nearly 200,000 uncultured viruses. We report the discovery of 2,628 putative endolysins, many of which displayed novel domain architectures. In addition, several of the identified proteins are predicted to be active against genera that include pathogenic bacteria. These discoveries enhance the diversity of known endolysins and are a stepping stone for developing medical and biotechnological applications that rely on bacteriophages, the most diverse biological entities on Earth.

## Introduction

Bacteriophages (Phages) have evolved a multitude of endolysins for the purpose of degrading the complex cell wall structures of their bacterial hosts (Pimentel, 2014). Despite sharing a common function, endolysins are a diverse class of enzymes with a multitude of action mechanisms and protein architectures (Oliveira et al., 2013; Vidová et al., 2014). Protein domains within endolysins grant them their host specificity and efficiency (Hermoso et al., 2007). Multiple classes of endolysins have been described and classified according to the specific enzymatic activities of their catalytic domains, including glycosidases, amidases, and carboxy/endo-peptidase (Schmelcher et al., 2012). Endolysins of phages that infect Gram-positive bacteria often have a modular structure that includes one enzymatic catalytic domain (ECD) at the N-terminal and at least one cell wall binding domain (CBD) at the C-terminal portion of the protein connected by a flexible interdomain linker (Nelson et al., 2012). Meanwhile endolysins of phages that infect Gram-negative bacteria often exhibit a globular structure that contains a single catalytic domain (Pohane and Jain, 2015).

Some endolysins can degrade the cell wall from the outside, which grants them potential to be used for biotechnological and medical applications (Nelson et al., 2012; Gutiérrez et al., 2018). The ongoing crisis of antibiotic based treatments calls for alternative strategies for fighting bacterial infections (Ventola, 2015) and endolysins have multiple advantages over antibiotics: they have higher target specificity and so far no forms of resistance have been reported (Nelson et al., 2012). Furthermore, endolysins can be engineered to alter their host range and efficiency (Díez-Martínez et al., 2014; Blázquez et al., 2016). Several recombinant endolysins are now in preliminary study phase for use in human and veterinary medicine, with promising results for the treatment against both Gram-positive and Gram-negative bacteria (Briers et al., 2014; Cooper et al., 2016). Some endolysins can even combat intracellular human pathogens (Shen et al., 2016), demonstrating that their potential applicabilities are wider than originally conceived. Finally, these enzymes can be cloned into expression vectors for large scale synthesis. Therefore the potential applications of endolysins are not only of medical but also of industrial (e.g., detecting food-borne pathogens) and agricultural relevance (e.g., treatment against phytopathogens) (Schmelcher and Loessner, 2016).

Despite the recognized diversity and potential of endolysins, our current understanding of these enzymes is limited. Analysis of sequence repositories suggests that less than 1,000 of these proteins are currently known (Oliveira et al., 2013). Although previous studies have characterized the diversity of endolysins through bioinformatic approaches, they focused on reference genomes of isolated phages and prophages, and overlooked environmental phages that have not yet been isolated or cultured (Oliveira et al., 2013; Vidová et al., 2014). Thus, currently available catalogs of endolysins do not cover the diversity of enzymes encoded in the genomes of the many uncultured phages spread across Earth’s many ecosystems. Culture independent approaches have revolutionized our understanding of phage genetic diversity, revealing thousands of phage genomes and entirely novel evolutionary lineages at an unprecedented scale (Mizuno et al., 2013; Roux et al., 2015; Paez-Espino et al., 2016; Yutin et al., 2017). These novel genomes are a rich resource for the discovery of endolysins that could have unique domain architectures and target hosts for which no endolysins are currently known. Thus, we sought to screen the genomes of bacteriophages discovered through culture independent approaches to expand the known repertoire of endolysins, asses their structural diversity, and determine how this diversity changes across targeted hosts and ecosystems.

## Materials and Methods

A database of uncultured viral genomes was compiled from publications aimed at large scale discovery of phages without culturing (Mizuno et al., 2013, 2016; Roux et al., 2015, 2016; Paez-Espino et al., 2016; Coutinho et al., 2017). This dataset comprised 183,298 genomic sequences (**Supplementary File S1**) of uncultured viral genomes adding up to 2.9 Gbp of raw data (**Supplementary Table S1**). We also retrieved available metadata associated with those sequences regarding the ecosystems from which they originated, and the predicted hosts reported in the original publications. These studies used multiple strategies to assign hosts to metagenomic contigs which included: high genomic similarity to reference phage genomes; homology matches between phage and prokaryotic genomes; CRISPR spacers from prokaryotic genomes matching metagenomic contigs; similarity between phage and prokaryotic tRNAs; and co-occurrence of phage genome pairs indicative of a shared host. Prophages described by Roux et al. (2015) were assigned hosts according to the prokaryotic genomes in which they were identified.

A dataset of *bona fide* endolysin sequences encoded in genomes of double stranded DNA phages was compiled to be used as a reference database. This database comprised 629 proteins from NCBI RefSeq phages and was manually curated so to exclude structural lysins (i.e., exolysins) (Oliveira et al., 2013). Prodigal (Hyatt et al., 2010) was run in metagenomic mode to identify protein encoding genes of uncultured phage genomes. All predicted protein sequences were queried against the reference endolysin database using Diamond (Buchfink et al., 2015). Proteins that had hits to the reference database were classified as putative endolysins if matches were within the following thresholds: identity ≥ 50%, e-value ≤ 0.001, query coverage ≥ 30%, and alignment length ≥ 50 amino acids. Protein domains of putative endolysins were identified by querying sequences against the Pfam database using HMMER version v3.1b2 (Finn et al., 2015) with default parameters. Additionally, putative endolysins were analyzed through SignalIP (Petersen et al., 2011) to detect signal peptide sequences. Finally, both the putative and reference endolysins were clustered into orthologous groups (OGs) using OrthoMCL (Li et al., 2003) within the GET_HOMOLOGUES pipeline (Contreras-Moreira and Vinuesa, 2013) by setting an inflation factor of 1 and all other parameters set to default. Multiple sequence alignments were constructed through Clustal Omega (Sievers et al., 2014) for each OG represented by at least 10 proteins. Alignments were used to perform phylogenetic reconstructions through FastTree (Price et al., 2010) using default parameters (Amino acid distances BLOSUM45 and Jones-Taylor-Thorton model and support value calculation). An additional tree was built based on a multiple alignment of all proteins assigned to OGs of 10 or more proteins.

## Results and Discussion

A total of 2,628 putative endolysins were identified (**Supplementary File S2**). Homolog identification clustered these proteins into 297 OGs. We focused downstream analysis on 46 OGs represented by 10 or more proteins. Each of these OGs was manually validated as true endolysins by inspecting for: presence of *bona fide* endolysins from the reference database; prevalence of typical endolysins domains (e.g., Lysozyme, Amidase, and Glycosyl Hydrolase); and close phylogenetic relationship between putative and *bona fide* endolysins as assessed by inspecting phylogenetic trees generated for each OG. Considering the degree of diversity among phage genomes and the rate in which their genes evolve, we used very conservative thresholds to identify putative endolysins (identity ≥ 50%, e-value ≤ 0.001, query coverage ≥ 30% and alignment length ≥ 50 amino acids). Yet, due to high levels of structural similarities between structural lysins and endolysins, it is possible that some of the putative endolysins identified are actually structural lysins (enzymes used by phages to breach the cell wall at the beginning of the infection process). Yet, using a curated database of *bona fide* endolysins from reference phage genomes, conservative thresholds, and manual validation of the OGs should minimize the occurrence of false positives in our dataset.

**FIGURE 1:**
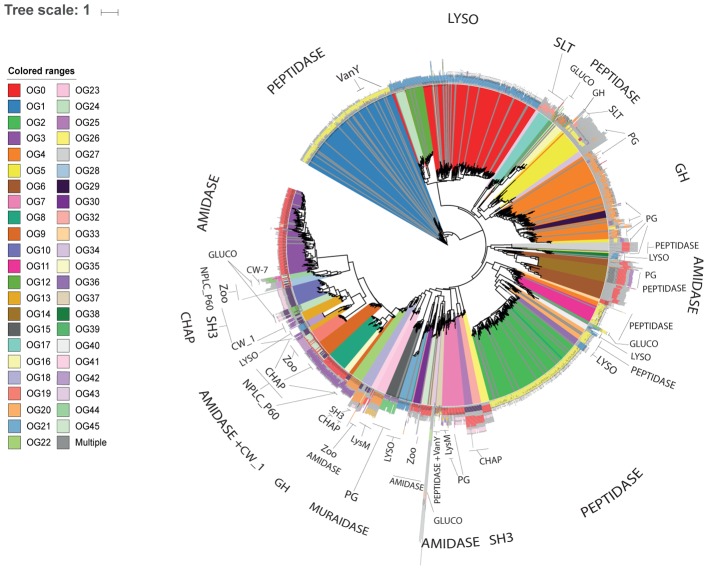
Novel diversity of endolysins discovered in uncultured phage genomes. Cladogram displaying the phylogenetic reconstruction of endolysins assigned to the 46 OGs represented by 10 or more proteins. Branches are colored according to OG assignments. Protein architecture is displayed adjacent to each leaf, identical colors represent identical Pfam domains.

Proteins assigned to the same OG often displayed identical domain architectures, although some exceptions were observed (**Figure 1** and **Supplementary File S3**). A total of 62 domains were identified across 46 OGs (**Table 1**), including 26 types of ECDs, 12 CBDs, and 24 domains of unknown function. For 27 OGs only a single catalytic domain was observed. Meanwhile, 19 OGs harbored at least one protein with both cell wall binding and ECDs. Signal peptide sequences were detected only in six OGs, suggesting that most of the discovered endolysins rely on other mechanisms for membrane translocation such as holin-dependent transportation. The most frequent domain observed in the putative endolysins was phage_lysozyme (PF00959) a Glycoside Hydrolase, followed by Peptidase_M15 (PF08291), and Amidase_2 (PF01510).

**Table 1:** Prevalence of domains, signal peptide and inferred cell wall target of endolysin orthologous groups.

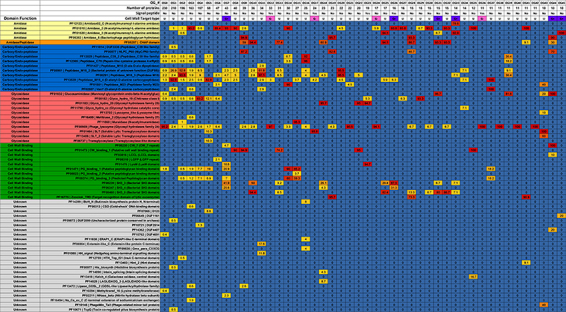

**FIGURE 2:**
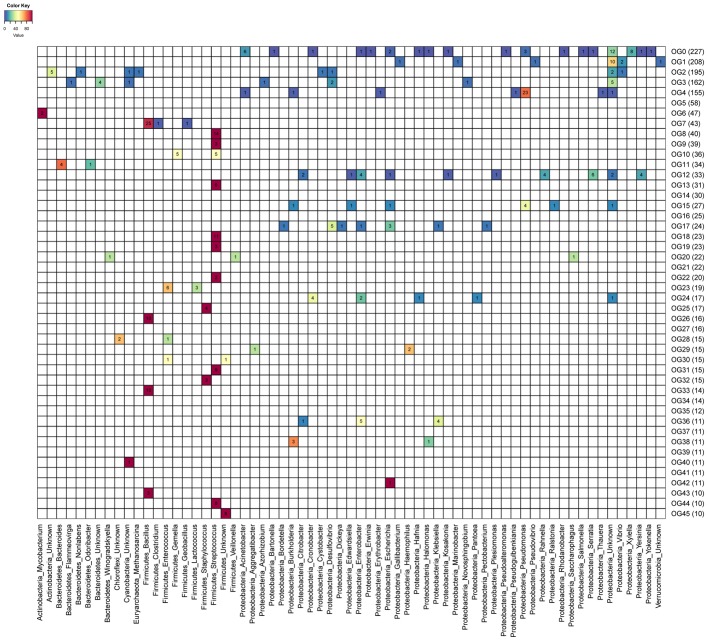
Heatmap depicting prevalence of targeted host genus across endolysin orthologous groups (OGs). Numbers within cells represent the amount of proteins targeting each host genus (columns) among the 46 OGs (rows) represented by 10 proteins or more. Cell colors depict the percentage frequency of each host among OGs. Calculated percentages do not include proteins derived from genomic sequences without host assignments.

We investigated associations between OGs and predicted hosts of uncultured phages. A total of 32 OGs had at least one endolysin derived from a phage genome for which host prediction was available. Endolysins from OGs predicted to target Gram-negatives were often composed of a single ECD, while those derived from OGs predicted to target Gram-positive bacteria were often composed of both a CBD and an ECD. Often a single bacterial genus could be targeted by multiple OGs (**Figure 2**). For example, enzymes targeting *Streptococcus* were present in nine distinct OGs and those targeting *Bacillus* were found in four OGs. Most OGs are predicted to be active against multiple bacterial genera with only two exceptions: OG44 and OG26 which respectively target *Streptococcus* and *Bacillus*. In general, OGs predicted to target multiple taxa were restricted to organisms with the same gram staining patterns. Interestingly endolysins from phages predicted to infect *Mycobacterium* were all assigned to a single group (OG6), likely due to the unique cell wall composition of these organisms.

A total of 34 OGs harbor at least one endolysin predicted to be active against genera that include potentially pathogenic bacteria (e.g., *Staphylococcus*, *Clostridium*, and *Klebsiella*). Ten of those OGs have at least one protein predicted to target bacteria currently classified by the World Health Organization as critical priority for the development of alternative therapies. In addition, five OGs include proteins predicted to be active against phytopathogens (e.g., *Xylella, Erwinina*, and *Burkholderia*) (Mansfield et al., 2012; Blomme et al., 2017). *In vivo* experiments are necessary to confirm the efficiency of these proteins for fighting pathogens, since different species or strains of bacteria that belong to the same genus may differ in their pathogenic potential.

**FIGURE 3:**
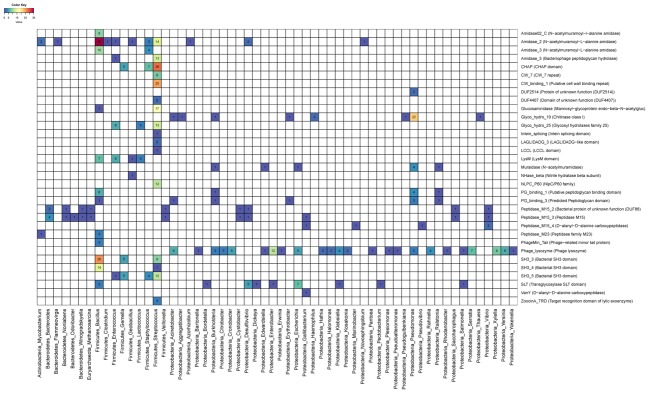
Heatmap depicting associations between targeted host genus and endolysin domains. Numbers and colors within cells represent the frequency of protein domains (rows) among endolysins targeting each host genera (columns).

Next we investigated the frequency of associations between endolysin domains and targeted bacteria (**Figure 3**). On the one hand, some genera were targeted by few domain categories. For example, most of the enzymes that targeted *Bacillus* have amidase (PF12123/PF01510/PF01520) activity in their catalytic domain and SH3 (PF08239/PF06347) on their CBDs, suggesting that this might be the most efficient mechanism to degrade the cell wall of the members of this clade. On the other hand, genera such as *Streptococcus* were targeted by endolysins that rely on multiple types of catalytic activities such as Amidase (PF01510/PF05382), Cysteine Histidine-dependent Amidohydrolases/Peptidases (CHAP) domain (PF05257), Glucosaminidase (PF01832), Glycosyl hydrolases family 25 (PF01183), as well as multiple CBDs such as CW1 (PF01473), CW7 (PF08230), and SH3 (PF08239/PF06347). This pattern suggests that multiple domain architectures might be efficient for targeting the cell walls of *Streptococcus*. Finally, the Phage_lysozyme (PF00959) and Soluble Lytic Transglycosylase (SLT, PF01464/PF13406) domains were often detected in endolysins predicted to target Proteobacteria, demonstrating that these domains are tuned to degrade the cell walls of this diverse phylum of Gram-negatives.

Host assignments for phage genomes derived from metagenomic datasets are based on bioinformatic predictions which vary on their degree of precision and recall. These approaches have high accuracy for higher taxonomic ranks such as phylum or class but sometimes lead to incorrect predictions at the lower ranks such as genus or species (Edwards et al., 2015; Coutinho et al., 2017). Thus, the associations between OGs/domains and targeted hosts inferred from metagenomic data should be interpreted as putative and in need of experimental validation (which is currently out of our scope). Yet the validity of our findings is corroborated by: (1) Use of conservative thresholds to maximize accuracy during the host prediction analysis described in the original publications that first reported phage genomes obtained from metagenomic samples. (2) Endolysin domains identified in uncultured phages match those previously described for isolated and cultured bacteriophages (Oliveira et al., 2013). (3) Approximately, 74% of host assignments obtained for the 46 OGs analyzed in depth were derived from prophage sequences integrated into host genomes that were obtained from pure cultures (instead of metagenomic data). Thus the bulk of the host/OG and host/domain associations are based on high confidence host assignments.

Several of the identified protein domains are not typically present in endolysins from reference phage genomes. Those include domains that are likely associated with peptidoglycan lysis such as Melibiase_2 (PF16499; Glycosyl hydrolases family 27) and Glyco_hydro_cc (PF11790; Glycosyl hydrolase catalytic core), but also domains with no obvious role in this process, such as: Methyltransf_16 (PF10294; Lysine methyltransferase), Lipase_GDSL_2 (PF13472; GDSL-like Lipase/Acylhydrolase family), and PhageMin_Tail (PF10145; Phage-related minor tail protein). Presence of the latter could be either the result of protein fusions or represent novel functions or action mechanisms of endolysins. Since computational domain identification may be subjected to errors, experimental validation is necessary to corroborate the presence of these domains and to determine their molecular functions. Nevertheless, our results demonstrate that the molecular versatility of endolysins is still poorly understood considering the lack of information available for so many of the identified domains.

Finally, we explored associations between endolysin architecture and their ecosystem of origin. Most of the putative endolysins were derived from aquatic ecosystems or human microbiome samples, which are the habitats most often sampled by metagenomic studies (**Supplementary Figure S1**). This pattern highlights the potential for endolysin discovery in these sites but also the need for investigations of other ecosystems such as plant associated and terrestrial habitats, that also yielded novel endolysins. Aquatic phages appear to be a rich resource for endolysins containing amidase, peptidase and glycosidase domains, which points to them as a source of endolysins active against both Gram-positive and Gram-negative bacteria. Meanwhile, endolysins derived from phages of the human microbiome most often have amidase and peptidase domains, typical of enzymes acting on Gram-positive hosts.

## Conclusion

We were able to notably expand the known diversity of endolysins and describe proteins with novel and often complex domain architectures, thus challenging the current understanding of the diversity of those enzymes. Our findings show that environmental phage genomes, specially those from aquatic and human associated microbiomes, are a rich resource for endolysins discovery. Since several of the identified endolysins are predicted to be effective against plant and animal pathogens, those are ideal candidates for purification and further characterization, specially those predicted to act on Gram-positive bacteria, against which endolysin therapy has showed the most promising results so far (Gerstmans et al., 2016). Our findings regarding associations between bacterial targets and domains provide insights for the engineering of recombinant proteins that have higher efficiency or an extended host spectrum. Further experimental research will be necessary to corroborate our findings regarding the endolytic activity, domain architecture, and target spectrum of these proteins and to evaluate their potential applications as antimicrobial agents. Culture independent approaches will continue to expand the genetic diversity of phages, and our strategy represents a simple, fast, and scalable approach for discovering endolysins encoded in their genomes.

## Author Contributions

IF-R, FC, and FR-V conceived the study and designed the experiments. IF-R and FC performed the experiments and analyzed the data. All authors contributed to writing the manuscript.

## Conflict of Interest Statement

The authors declare that the research was conducted in the absence of any commercial or financial relationships that could be construed as a potential conflict of interest.

## Supplementary Material

FIGURE S1 Heatmap depicting associations between ecosystem sources and endolysin domains. Numbers and colors within cells represent the frequency of protein domains (rows) among endolysins identified in phage genomes from each ecosystem category (columns).

TABLE S1 Detailed metadata of the genomic sequences of uncultured phages analyzed in this study: including sequence identifier, original dataset, sequence length, number of identified protein encoding genes, ecosystem source and host taxonomic classification.

FILE S1 Multifasta file containing the 183,6298 genomic sequences of uncultured viruses analyzed in this study. File can be downloaded here: https://drive.google.com/open?id=15rRJMfguPGhfiuQor7puIrlIr2J02tJQ

FILE S2 Multifasta file containing the amino acid sequences of 2,628 putative endolysins identified in the genomes of uncultured viruses.

FILE S3 Newick format file of the phylogenetic reconstruction of endolysin proteins displayed in **Figure 1**.
