# Supplementary material for: Thousands of Novel Endolysins Discovered in Uncultured Phage Genomes
Source: Front Microbiol. 2018 May 18;9:1033. doi: 10.3389/fmicb.2018.01033 (PMC5968864; doi:10.3389/fmicb.2018.01033)
Supplement: FIGURE S1 — Heatmap depicting associations between ecosystem sources and endolysin domains. Numbers and colors within cells represent the frequency of protein domains (rows) among endolysins identified in phage genomes from each ecosystem category (columns). [file Image_1.PDF]

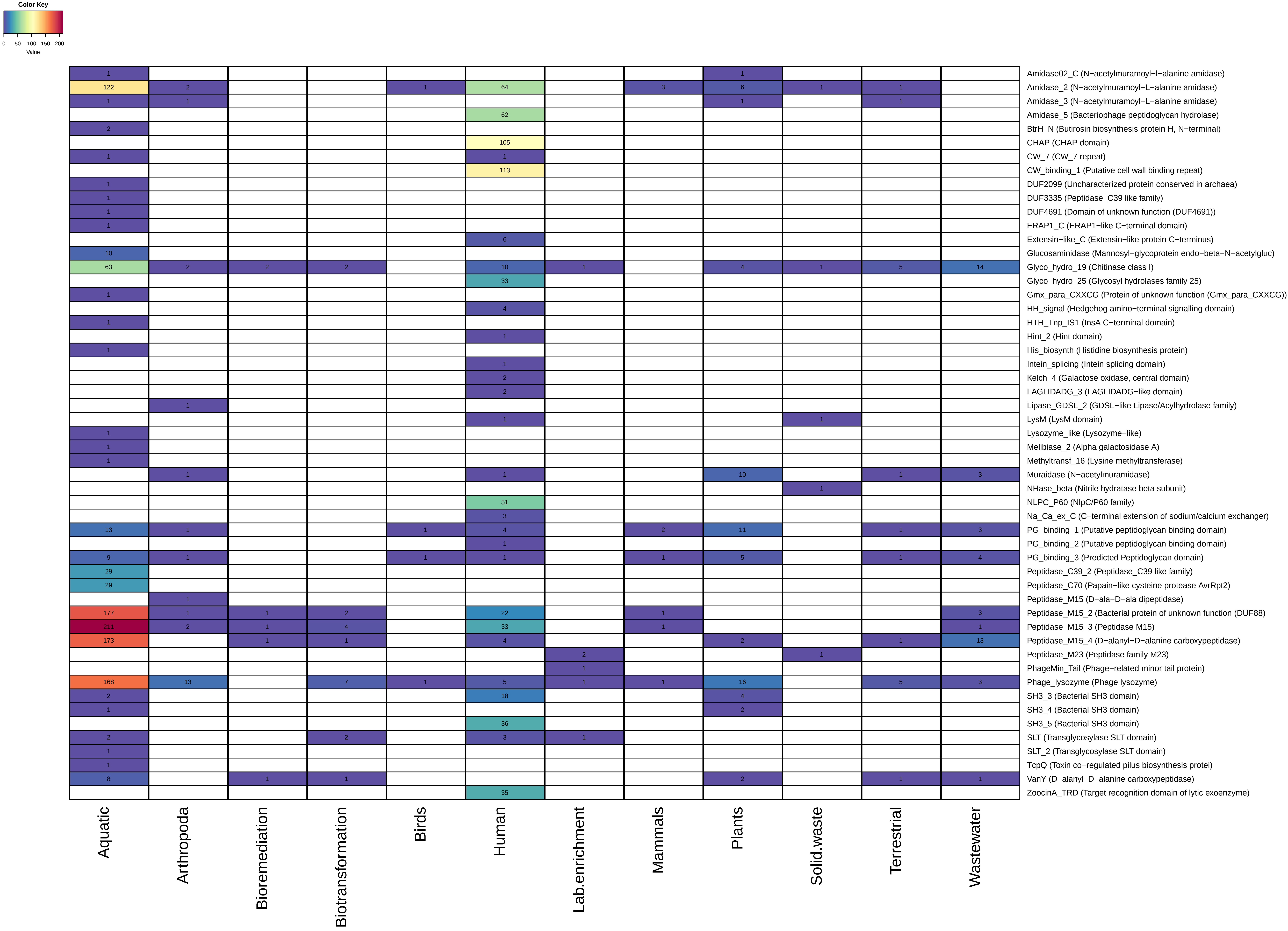

Color Key
0
50
100
150
200
Value
Amidase02_C (N−acetylmuramoyl−l−alanine amidase)
Amidase_2 (N−acetylmuramoyl−L−alanine amidase)
Amidase_3 (N−acetylmuramoyl−L−alanine amidase)
Amidase_5 (Bacteriophage peptidoglycan hydrolase)
BtrH_N (Butirosin biosynthesis protein H, N−terminal)
CHAP (CHAP domain)
CW_7 (CW_7 repeat)
CW_binding_1 (Putative cell wall binding repeat)
DUF2099 (Uncharacterized protein conserved in archaea)
DUF3335 (Peptidase_C39 like family)
DUF4691 (Domain of unknown function (DUF4691))
ERAP1_C (ERAP1−like C−terminal domain)
Extensin−like_C (Extensin−like protein C−terminus)
Glucosaminidase (Mannosyl−glycoprotein endo−beta−N−acetylgluc)
Glyco_hydro_19 (Chitinase class I)
Glyco_hydro_25 (Glycosyl hydrolases family 25)
Gmx_para_CXXCG (Protein of unknown function (Gmx_para_CXXCG))
HH_signal (Hedgehog amino−terminal signalling domain)
HTH_Tnp_IS1 (InsA C−terminal domain)
Hint_2 (Hint domain)
His_biosynth (Histidine biosynthesis protein)
Intein_splicing (Intein splicing domain)
Kelch_4 (Galactose oxidase, central domain)
LAGLIDADG_3 (LAGLIDADG−like domain)
Lipase_GDSL_2 (GDSL−like Lipase/Acylhydrolase family)
LysM (LysM domain)
Lysozyme_like (Lysozyme−like)
Melibiase_2 (Alpha galactosidase A)
Methyltransf_16 (Lysine methyltransferase)
Muraidase (N−acetylmuramidase)
NHase_beta (Nitrile hydratase beta subunit)
NLPC_P60 (NlpC/P60 family)
Na_Ca_ex_C (C−terminal extension of sodium/calcium exchanger)
PG_binding_1 (Putative peptidoglycan binding domain)
PG_binding_2 (Putative peptidoglycan binding domain)
PG_binding_3 (Predicted Peptidoglycan domain)
Peptidase_C39_2 (Peptidase_C39 like family)
Peptidase_C70 (Papain−like cysteine protease AvrRpt2)
Peptidase_M15 (D−ala−D−ala dipeptidase)
Peptidase_M15_2 (Bacterial protein of unknown function (DUF88)
Peptidase_M15_3 (Peptidase M15)
Peptidase_M15_4 (D−alanyl−D−alanine carboxypeptidase)
Peptidase_M23 (Peptidase family M23)
PhageMin_Tail (Phage−related minor tail protein)
Phage_lysozyme (Phage lysozyme)
SH3_3 (Bacterial SH3 domain)
SH3_4 (Bacterial SH3 domain)
SH3_5 (Bacterial SH3 domain)
SLT (Transglycosylase SLT domain)
SLT_2 (Transglycosylase SLT domain)
TcpQ (Toxin co−regulated pilus biosynthesis protei)
VanY (D−alanyl−D−alanine carboxypeptidase)
ZoocinA_TRD (Target recognition domain of lytic exoenzyme)
Aquatic
Arthropoda
Bioremediation
Biotransformation
Birds
Human
Lab.enrichment
Mammals
Plants
Solid.waste
Terrestrial
Wastewater
